# Anti-adhesion barrier gels following operative hysteroscopy for treating female infertility: a systematic review and meta-analysis

**DOI:** 10.1007/s10397-014-0832-x

**Published:** 2014-03-14

**Authors:** Jan Bosteels, Steven Weyers, Ben W. J. Mol, Thomas D’Hooghe

**Affiliations:** 1 Department of Obstetrics and Gynaecology, Imeldahospitaal, Imeldalaan 9, 2820 Bonheiden, Belgium; 2 CEBAM, Centre for Evidence-based Medicine, the Belgian Branch of the Dutch Cochrane Centre, ACHG, Kapucijnenvoer 33, blok J bus 7001, 3000 Leuven, Belgium; 3 Universitaire Vrouwenkliniek, University Hospital Gent, De Pintelaan 185, 9000 Gent, Belgium; 4 School of Paediatrics and Reproductive Health, The Robinson Institute, University of Adelaide, 5000 SA Adelaide, Australia; 5 Leuven University Fertility Centre, KU Leuven, University Hospital Gasthuisberg, Herestraat 49, 3000 Leuven, Belgium

**Keywords:** Adhesion prevention, Barrier gel, Operative hysteroscopy, Infertility, Systematic review, Meta-analysis

## Abstract

The aim of this study was to assess the effects of any anti-adhesion barrier gel used after operative hysteroscopy for treating infertility associated with uterine cavity abnormalities. Gynecologists might use any barrier gel following operative hysteroscopy in infertile women for decreasing de novo adhesion formation; the use of any barrier gel is associated with less severe de novo adhesions and lower mean adhesion scores. Nevertheless, infertile women should be counseled that there is at the present no evidence for higher live birth or pregnancy rates. There is a lack of data for the outcome miscarriage. Preclinical studies suggest that the use of biodegradable surgical barriers may decrease postsurgical adhesion formation. Observational studies in the human report conflicting results. We searched the Cochrane Menstrual Disorders and Subfertility Specialized Register (10 April 2013), the Cochrane Central Register of Controlled Trials (*The Cochrane Library* 2013, Issue 1), MEDLINE (1950 to 4 April 2013), EMBASE (1974 to 4 April 2013), and other electronic databases of trials including trial registers, sources of unpublished literature, and reference lists. We handsearched the *Journal of Minimally Invasive Gynecology* (from 1 January 1992 to 13 April 2013); we also contacted experts in the field. We included the randomized comparisons between any anti-adhesion barrier gel versus another barrier gel, placebo, or no adjunctive therapy following operative hysteroscopy. Primary outcomes were live birth rates and de novo adhesion formation at second-look hysteroscopy. Secondary outcomes were pregnancy and miscarriage rates, mean adhesion scores, and severity of adhesions at second-look hysteroscopy. Two authors independently assessed eligible studies for inclusion and risk of bias, and extracted data. We contacted primary study authors for additional information or other clarification. Five trials met the inclusion criteria. There is no evidence for an effect favoring the use of any barrier gel following operative hysteroscopy for the key outcomes of live birth or clinical pregnancy (risk ratio (RR) 3.0, 95 % confidence interval (CI) 0.35 to 26, *P* = 0.32, one study, 30 women, very low quality evidence); there were no data on the outcome miscarriage. The use of any gel following operative hysteroscopy decreases the incidence of de novo adhesions at second-look hysteroscopy at 1 to 3 months (RR 0.65, 95 % CI 0.45 to 0.93, *P* = 0.02, five studies, 372 women, very low quality evidence). The number needed to treat to benefit is 9 (95 % CI 5 to 33). The use of auto-cross-linked hyaluronic acid gel in women undergoing operative hysteroscopy for fibroids, endometrial polyps, or uterine septa is associated with a lower mean adhesion score at second-look hysteroscopy at 3 months (mean difference (MD) −1.44, 95 % CI −1.83 to −1.05, *P* < 0.00001, one study, 24 women; this benefit is even larger in women undergoing operative hysteroscopy for intrauterine adhesions(MD −3.30, 95 % CI −3.43 to −3.17, *P* < 0.00001, one study, 19 women). After using any gel following operative hysteroscopy, there are more American Fertility Society 1988 stage I (mild) adhesions (RR 2.81, 95 % CI 1.13 to 7.01, *P* = 0.03, four studies, 79 women). The number needed to treat to benefit is 2 (95 % CI 1 to 4). Similarly there are less’ moderate or severe adhesions’ at second-look hysteroscopy (RR 0.25, 95 % CI 0.10 to 0.67, *P* = 0.006, four studies, 79 women). The number needed to treat to benefit is 2 (95 % CI 1 to 4) (all very low quality evidence). There are some concerns for the non-methodological quality. Only two trials included infertile women; in the remaining three studies, it is not clear whether and how many participants suffered from infertility. Therefore, the applicability of the findings of the included studies to the target population under study should be questioned. Moreover, only one small trial studied the effects of anti-adhesion barrier gels for the key outcome of pregnancy; the length of follow-up was, however, not specified. More well-designed and adequately powered randomized studies are needed to assess whether the use of any anti-adhesion gel affects the key reproductive outcomes in a target population of infertile women.

## Background

Intrauterine adhesions (IUAs) are fibrous strings at opposing walls of the uterus. The spectrum of IUA formation may vary from minimal IUAs to the complete obliteration of the uterine cavity. The causes of IUAs are multifactorial; nearly 90 % of cases are associated with postpartum or postabortion dilatation and curettage. The role of infection in the development of IUAs is controversial with the exception of genital tuberculosis [1]. The pathophysiology and the mechanisms of tissue repair in the endometrium are moreover poorly understood despite several theories on the source of cells for human endometrial regeneration [2].

**Fig. 1:**
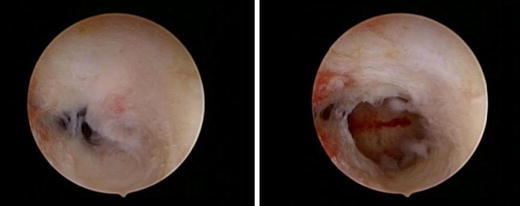
Intrauterine adhesions

IUA formation is the major long-term complication of operative hysteroscopy in women of reproductive age (Fig. 1). According to a randomized controlled trial (RCT) on the effectiveness of preoperative treatment before operative hysteroscopy, the incidence of postsurgical IUAs at second-look hysteroscopy is 3.6 % after polyp removal, 6.7 % after resection of uterine septa, 31.3 % after removal of a single fibroid, and 45.5 % after resection of multiple fibroids [3]. The investigators of a prospective cohort study in 163 women undergoing operative hysteroscopy conclude that the duration of the endometrial wound healing differs according to the type of pathology treated [4]. At follow-up hysteroscopy 1 month after the surgical intervention, significantly more women achieve a full healing of the endometrial cavity after removal of endometrial polyps (32 of 37 women or 86 %) compared to hysteroscopic lysis of intrauterine adhesions (30 of 45 women or 67 %), treatment of uterine septum (three of 16 women or 19 %), or removal of submucous fibroids (12 of 65 women or 18 %) (*P* < 0.05). Significantly more de novo IUAs are detected in women undergoing septoplasty (14 of 16 women or 88 %) or adhesiolysis (34 of 45 women or 76 %) compared to removal of submucous fibroids (26 of 65 women or 40 %) or endometrial polyps (zero of 37 women or 0 %). Women with de novo IUAs are less likely to achieve full endometrial wound healing within 1 month compared with those without de novo IUAs (23 of 74 women or 31 % versus 54 of 89 women or 61 %, *P* = 0.0003). The authors conclude that the full recovery of the endometrium varies from 1 month after the removal of polyps to between 2 and 3 months following hysteroscopic myomectomy [4].

Intrauterine adhesions may cause poor reproductive outcome. Firstly, according to a large review of observational studies, 922 of 2,151 women with IUAs (43 %) suffer from infertility [5]. The hypothetical underlying mechanisms for infertility due to IUAs are obstruction of sperm transport into the cervix, impaired embryo migration within the uterine cavity, or failure of embryo implantation due to endometrial insufficiency [1]. Secondly, recurrent miscarriage is often associated with IUAs; the prevalence of IUAs in women suffering from this health problem ranges from 5 to 39 % according to a narrative review of observational studies [6]. Thirdly, the hysteroscopic treatment of severe IUAs may cause long-term major obstetrical complications, such as placenta accreta/increta and higher risks for preterm delivery, uterine rupture, and postpartum hysterectomy [1].

Hyaluronic acid or hyaluronan (HA) is a water-soluble polysaccharide: It consists of multiple disaccharide units of glucuronic acid and *N*-acetylglucosamine, bound together by a β1-3-type glucoside bond. Solutions of HA have interesting viscoelastic properties which have led to interests in developing applications of HA in surgical procedures, for example in eye surgery. HA is not an ideal substance for all procedures, due to its limited residence time when applied to a surgical site. It quickly enters the systemic circulation and is cleared rapidly by catabolic pathways. Attempts to use hyaluronan for preventing postsurgical adhesions have therefore been met with variable success. Several chemically modified derivatives of HA have been developed to circumvent the disadvantages of HA. One such derivative is auto-cross-linked polysaccharide (ACP). It is formed by cross-linking hyaluronan, by direct formation of covalent ester bonds between hydroxyl and carboxyl groups of the hyaluronan molecule. ACP can be prepared with various degrees of cross-linking, which allows tailoring of the viscosity properties of ACP gels [7]. Carboxymethylcellulose (CMC) is a high molecular weight polysaccharide that has a viscosity greater than Dextran 70. CMC can be used for adhesion prevention as a membrane barrier or a gel as a mixture of chemically derivative sodium hyaluronate and carboxymethylcellulose gel (HA–CMC) [8].

The ideal anti-adhesion barrier following operative hysteroscopy would be the application of a biologically active mechanical separator that achieves the suppression of intrauterine adhesion formation and promotes the healing of the endometrial tissue. The use of the biodegradable gel surgical barriers is based on the principle of keeping the adjacent wound surfaces as mechanically separate [7]. Several preclinical studies in various animal models report the effectiveness of both ACP [9–16] and HA–CMC gels [8, 17] or HA-CMC membranes [18, 19] for preventing postsurgical adhesions. Other preclinical studies in animal models suggest that HA gel remains in situ for more than 5 to 6 days [20, 21]. Similarly, animal studies demonstrate the persistence of HA–CMC for about 7 days after its application [22]. However, most of these studies were done in rodent models, and not in nonhuman primate models with reproductive anatomy similar to humans, like the baboon, a validated model for endometriosis research [23]

The exact mechanisms by which ACP and HA–CMC are able to reduce adhesion reformation are not well known, but may be related to “hydroflotation” or “siliconizing” effects. One French clinical controlled trial (*N* = 54 women) studied the effectiveness of the application of ACP gel (*n* = 30) versus no gel (*n* = 24) at the end of an operative hysteroscopic procedure for treating fibroids, polyps, uterine septa, or IUAs; there are no statistically significant differences for the rate of adhesion formation, the mean adhesion scores, or the severity of the adhesions between both comparison groups [24]. No data are available for the reproductive outcome.

The health burden associated with infertility, abdominal pain, or bowel obstruction due to adhesions is substantial [7, 25, 26]; the total cost of adhesion-related morbidity in the US Health Care system exceeds $1 billion annually [27]. To the best of our knowledge, no economical studies on adhesion prevention after operative hysteroscopy have been conducted in an infertile population.

Postoperative de novo adhesion formation is a determining factor influencing endometrial wound healing [4]. At the present, there is uncertainty whether the use of anti-adhesion barrier gels following operative hysteroscopy affects the pregnancy or live birth rates; this is the main objective of the present systematic review.

## Methods

Two reviewers independently searched the Cochrane Menstrual Disorders and Subfertility Specialized Register (10 April 2013), the Cochrane Central Register of Controlled Trials (*The Cochrane Library* 2013, Issue 1), MEDLINE (1950 to 4 April 2013), and EMBASE (1974 to 4 April 2013) using a combination of both index and free-text terms. We used no language restrictions. We searched other electronic databases of trials including trial registers, sources of unpublished literature, and reference lists. We handsearched the *Journal of Minimally Invasive Gynecology* (from 1 January 1992 to 13 April 2013) and contacted experts in the field.

We included only studies that were clearly randomized or claimed to be randomized. Studies were selected if the source population included women of reproductive age suffering from infertility, bound to undergo operative hysteroscopy for suspected or unsuspected intrauterine pathology before spontaneous conception or any infertility treatment. Infertility was defined as “a disease of the reproductive system defined by the failure to achieve a clinical pregnancy after 12 months or more of regular unprotected sexual intercourse” [28]. Studies were excluded if infertility was explicitly reported among the exclusion criteria.

We included the following types of randomized comparisons: any anti-adhesion barrier gel versus placebo, no barrier gel, or another type of barrier gel following operative hysteroscopy. We did not include studies of other anti-adhesion therapies, such as the use of human amnion membrane grafting, insertion of a balloon catheter or IUD, or hormonal treatment; this review focuses exclusively on the effectiveness of anti-adhesion barrier gels.

We selected live birth and de novo adhesion formation at second-look hysteroscopy as primary outcomes. Live birth was defined as a delivery of a live fetus after 20 completed weeks of gestational age that resulted in at least one live baby born. The delivery of a singleton, twin, or multiple pregnancy was counted as one live birth [28]. Ongoing or clinical pregnancy, miscarriage, and mean adhesion scores or severity of adhesions at second-look hysteroscopy were secondary outcomes. Ongoing pregnancy was defined as a pregnancy surpassing the first trimester or 12 weeks of pregnancy; clinical pregnancy was defined as a pregnancy diagnosed by US visualization of one or more gestational sacs or definitive clinical signs of pregnancy [28]. There are at the present seven reported classification systems for scoring the extent or severity of intrauterine adhesions [1]. Some classification systems have incorporated menstrual and obstetric history [29–31]; others rely exclusively on the hysteroscopic evaluation of the uterine cavity [32–35]. None of these systems has been validated or universally accepted [1]. We avoided pooling data from studies using different scoring systems.

One reviewer screened the titles and abstracts from the search to remove the publications which were obviously irrelevant for the research question of the present systematic review. After removing duplicates and after linking multiple reports of the same study together, two reviewers independently assessed the studies by examining the full text reports. This was done without blinding them to the reviewers; studies that appeared to be eligible were included using a pretested data extraction form. We contacted the authors of the primary study report whenever additional information was required. For studies with multiple study reports, we used the main trial report as the primary data extraction source.

Two reviewers independently assessed the risk of bias of the included studies and across studies by using the Cochrane “Risk of bias” tool. The following six items were assessed: random sequence generation, allocation concealment, blinding of participants and personnel, blinding of outcome assessors, selective outcome reporting, and other potential sources of bias. Any disagreements between the reviewers for the selection, data extraction, or risk of bias assessment were resolved through arbitration by a third author; any residual disagreement was reported in the final review.

We used the numbers of events in the comparison groups of each study to calculate the Mantel–Haenszel risk ratios (RR) for the binary data for all the main outcomes; for the secondary outcome “adhesion scores,” the mean values and the standard deviations (SD) were used to calculate the inverse variance mean differences (MD) and the 95 % confidence intervals (CI). We used the most recently updated Review Manager 5 software provided by the Cochrane Collaboration for all the calculations, including the 95 % CI.

All main outcomes were expressed as per woman randomized. Multiple live births and multiple pregnancies were counted as one event. We did not attempt to pool any reported data that did not allow a valid analysis, such as “per cycle” data.

We aimed to analyze the data on an intention-to-treat basis (ITT). We tried to obtain as frequently as possible missing data after contacting the primary study authors. If missing data could not be obtained, we undertook imputation of individual values for the primary outcomes only by assuming that live births or de novo adhesions would not have occurred in participants without a reported primary outcome. For all other main outcomes, we used an available data analysis. We subjected any imputation of missing data for the primary outcomes to sensitivity analyses; any substantial difference in the imputed ITT analyses compared to available data analyses was incorporated in the interpretation of the study findings and the discussion.

Meta-analysis was done to provide a meaningful summary whenever enough studies which were sufficiently similar with respect to the clinical and methodological characteristics were available. A formal assessment of statistical heterogeneity was done by using the *Q* statistic and the *I* ^2^ statistic; the combination of both tests is more sensitive to detect the likelihood of substantial statistical heterogeneity. A low *P* value of the *Q* statistic (*P* < 0.10) means significant heterogeneous results among individual studies. The *I* ^2^ statistic describes the percentage of variation across studies that is caused by substantial statistical heterogeneity rather than random chance variation; an *I* ^2^ statistic >50 % is the cutoff above which substantial statistical heterogeneity might be present. If there was evidence of substantial heterogeneity, we aimed to explore possible explanations for this observed heterogeneity by performing sensitivity analyses using Review Manager 5 software.

Publication bias, reporting bias, and within-study reporting bias are difficult to detect and correct for. We aimed to do the search for eligible studies as comprehensively as possible and by being alert in identifying duplicated reports of trials in order to minimize the potential impact of reporting and publication bias. Since we retrieved only a limited number of studies, we did not study publication bias or other forms of small study effects by creating a funnel plot.

One reviewer entered the study data and carried out the statistical analysis using Review Manager 5. We considered the outcomes live birth and pregnancy to be positive outcomes of effectiveness and by consequence higher numbers of these events as a benefit. The outcomes miscarriage, de novo adhesion formation, and adhesion scores were on the contrary considered as negative outcomes and higher numbers as harmful. We planned to combine data from primary studies in a meta-analysis with Review Manager 5 using the risk ratio as a summary outcome measure using a random-effects model if enough studies were retrieved and after significant clinical diversity and substantial statistical heterogeneity were confidently ruled out.

We planned to carry out subgroup analyses according to the extent or the severity of the uterine abnormality treated and for studies that reported both “live birth” and “pregnancy” in order to assess any overestimation of the treatment effect. We planned to do sensitivity analyses for the primary outcomes to investigate whether the results and conclusions are robust to arbitrary decisions regarding the eligibility and analysis. These sensitivity analyses included consideration whether conclusions would have differed if the eligibility was restricted to studies without high risk of bias versus all studies or if alternative imputation strategies were adopted, e.g., using odds ratio rather than risk ratio for the summary effect measure or a fixed effect rather than a random effects as the analysis model.

## Findings

### Description of studies

#### Results of the search

**Fig. 2:**
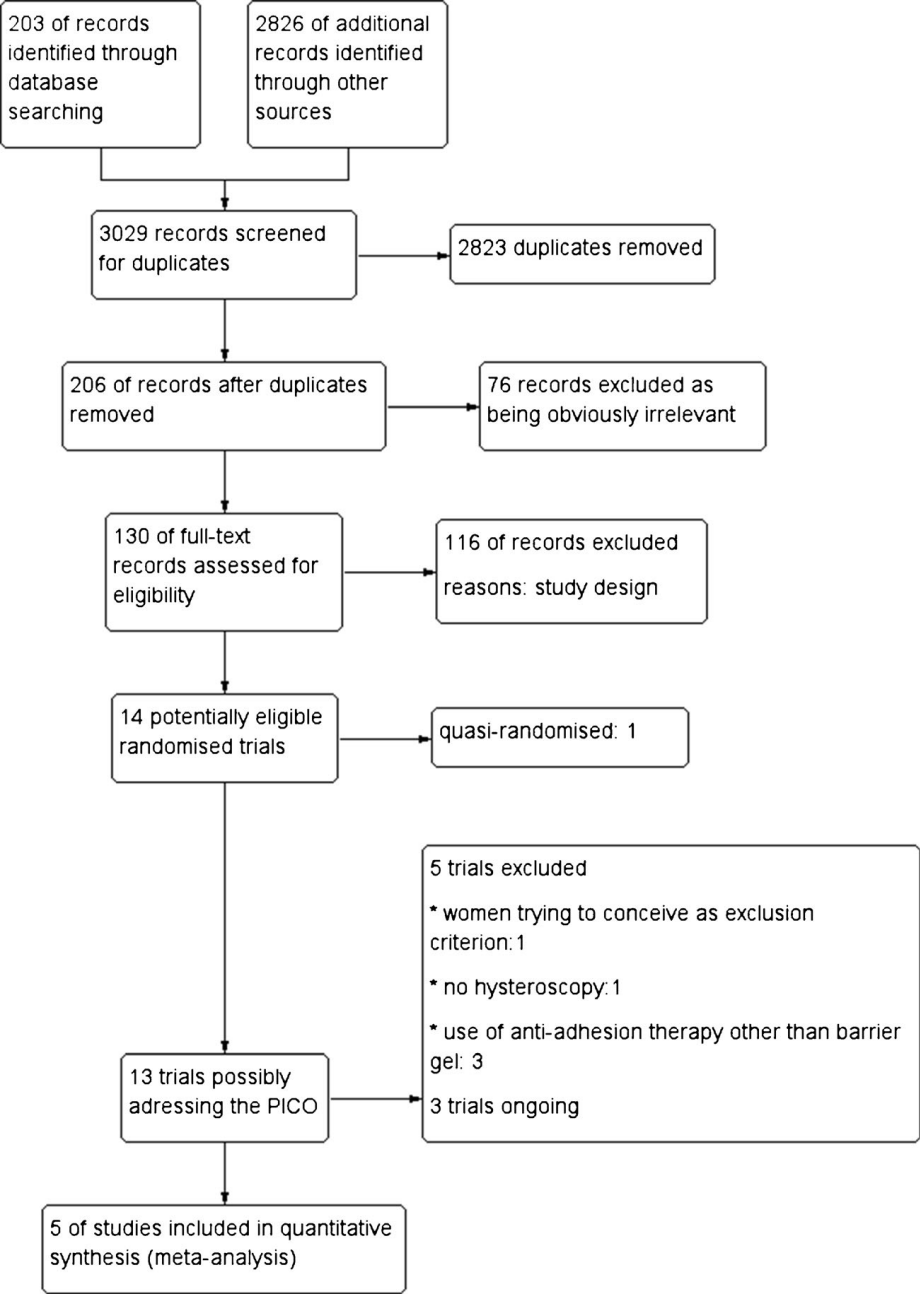
Study flow diagram

We identified 203 citations from searching electronic databases. These were combined with 2,826 additional records from other resources. We screened 3,029 records for duplicates by using End Note Web 3.5 and removed 2,823 duplicate citations. The remaining 206 records were assessed for eligibility through checking the titles and/or abstracts. We excluded 76 records as being obviously irrelevant. The remaining 130 full-text articles were assessed for eligibility. We retrieved 14 potentially eligible randomized studies; we included five trials, six trials were excluded, and three are ongoing. See Fig. 2 for the PRISMA flow chart of the search and selection process.

#### Included studies

##### Study design and setting

Five single-center parallel group RCTs were included in the present systematic review: Four were conducted in Italy [36–39] and one in Israel [40]. All five trials used two comparison groups.

Only one trial [38] reported a statistical power calculation for one of the primary outcomes (incidence of de novo adhesion formation). The protocol of all included trials was approved by the Institutional Review Board. The protocol of the trial from Israel [40] was registered in a clinical trial registry (see NCT01377779 in Clinical Trials.gov). None of the trials reported on funding or other potential conflicts of interest.

##### Participants

Two of the four Italian trials included infertile women: 34 women out of 92 participants [36] and 21 out of 110 participants [38]. The characteristics and data from these infertile women were not available for individual patient data meta-analysis. In the remaining two studies including 60 women [37] and 138 women [39], it is not clear whether and how many participants suffered from infertility. Regrettably we could not obtain any further clarification from the study authors. The study from Israel included 30 women who were trying to conceive after miscarriage; the proportion of women suffering from infertility was, however, not reported, and this could not be clarified either [40].

Three of the four Italian trials [36, 38, 39] were conducted in the same university hospital; several co-authors participated in the clinical research of all these trials. The in- and exclusion criteria were very similar in these three studies; one trial included only women with intrauterine adhesions [36]; the other trials included women with fibroids, polyps, or uterine septa [39] or women with single or multiple intrauterine lesions except intrauterine adhesions or suffering from dysfunctional uterine bleeding [38]. The description of the source population was not adequate in the fourth Italian study [37]. The fifth included study was conducted in a source population of women with retained products of conception after miscarriage [40].

The mean age of the participants was below 35 years in one study [36]. In two trials, the mean patient age in both comparison groups was above 35 years [38, 39]. The other two studies reported a range between 18 and 65 years [37] or 18 and 50 years [40] without reporting data on the mean ages and SD in both comparison groups.

##### Interventions

Five trials studied the randomized comparison between the intrauterine application of an anti-adhesion gel and no gel following operative hysteroscopy. In three studies, auto-cross-linked hyaluronic acid gel was used [36, 37, 39]; the other two [38, 40] used polyethylene oxide–sodium carboxymethylcellulose gel for the intervention. In three trials, the gel was administered into the uterine cavity through one of the flow channels of the resectoscope; the procedure was judged to be adequate when under hysteroscopic control the gel seemed to have replaced the liquid medium, filling the cavity from the fundus to the internal ostium of the cervix [36, 38, 39]. In one of these three studies [36], ultrasonographic data demonstrated that the anti-adhesive gel was able to keep the uterine walls separated for at least 72 h. In one study [37], the gel was applied using the cannula in a blind way without using hysteroscopic vizualization; for another trial [40], the method of application of the anti-adhesion gel is not clear.

##### Outcomes

The primary outcome of live birth was reported in none of the included studies; the incidence of de novo adhesions was reported in all five studies [36–40]. The following secondary outcomes were reported as follows: clinical pregnancy [40], mean adhesion scores [36, 39], and severity of the adhesions [36–40]. The definition of pregnancy and the time period during which this secondary outcome was assessed in one trial [40] was not described. Four studies [36, 38–40] used the 1988 American Fertility Society (AFS) classification system for scoring intrauterine adhesions at second-look hysteroscopy; one trial [37] used the ASRM modified scoring system. None of these two classifications has been validated since to the best of our knowledge neither of them has been directly linked to reproductive outcome. In all five studies, the incidence and the severity of adhesion formation outcomes were measured at one time point only, ranging from 4 to 12 weeks after the operative hysteroscopy [36–40].

### Risk of bias in included studies

#### Allocation (selection bias)

We judged four of the five trials to be at low risk for selection bias related to random sequence generation [36–39]. One trial [40] did not describe the method of random sequence generation; no further clarification could be obtained. We judged all five studies to be at unclear risk for selection bias related to allocation since they did not adequately describe the method of allocation concealment [36–40].

#### Blinding (performance bias and detection bias)

In all five trials, the method of blinding of the outcome assessors was not described [36–40]. We judged this risk of bias item to be important for the outcomes incidence of de novo adhesions, mean adhesion scores, and severity of adhesions but less relevant for the outcomes of live birth, ongoing or clinical pregnancy, and miscarriage unless the follow-up period was not long enough.

#### Incomplete outcome data (attrition bias)

We judged four trials to be at low risk for attrition bias [36, 38–40]. We judged one study to be at high risk for attrition bias related to incomplete outcome data; the loss to follow-up in this study of 33 % (20 out of 60 enrolled women) is sufficiently high and thus very likely to cause substantial attrition bias [37].

#### Selective outcome reporting (reporting bias)

We judged all trials to be at low risk of reporting bias; no evidence for selective outcome reporting was retrieved in any of the included studies when comparing abstract, methods, and results section [36–40].

#### Other potential sources of bias

**Fig. 3:**
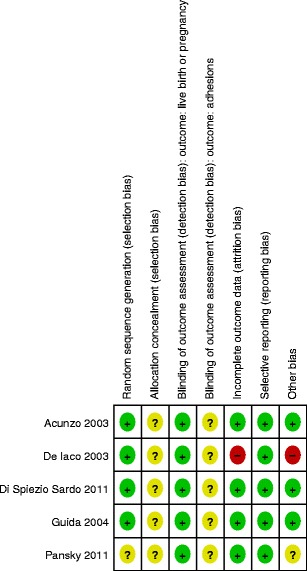
Risk of bias summary: review authors’ judgments about each risk of bias item for each included study

**Fig. 4:**
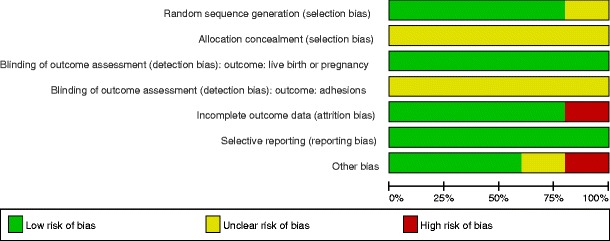
Risk of bias graph: review authors’ judgments about each risk of bias item presented as percentages across all included studies

We judged three studies to be at low risk for other potential sources of bias [36, 38, 39]. We judged one study to be at an unclear risk for other potential sources of bias [40]; the other study [37] was judged to be at high risk of bias due to likely imbalance of patient characteristics, imbalanced distribution of co-treatment, and other methodological study flaws (Figs. 3 and 4).

### Effects of interventions

#### Any gel versus no gel

##### Primary outcomes

**Fig. 5:**
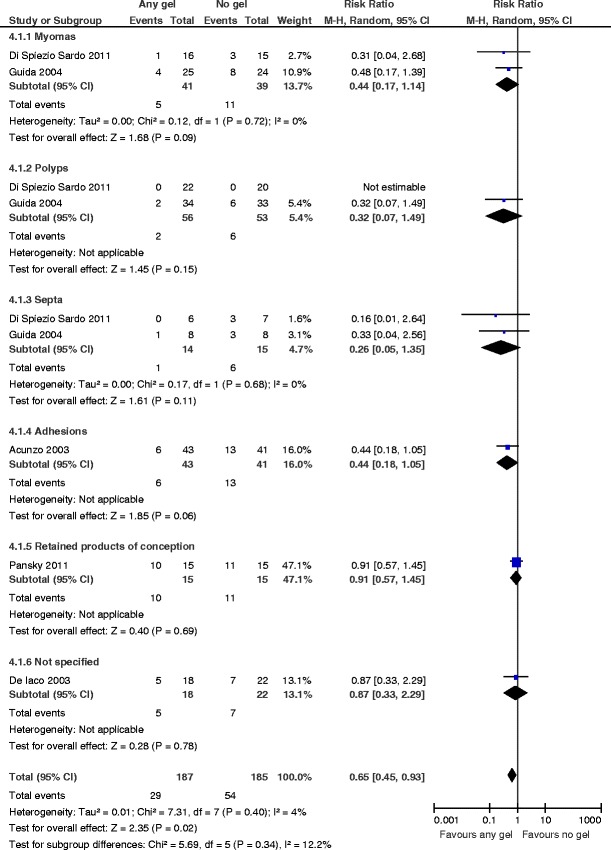
Any anti-adhesion gel versus no gel, outcome 2: incidence of de novo adhesions at second-look hysteroscopy

1. Live birth
  There were no data for this primary outcome.
2. Incidence of de novo adhesion formation at second-look hysteroscopy
  The use of any gel following operative hysteroscopy decreases the incidence of de novo adhesions (RR 0.65, 95 % CI 0.45 to 0.93, *P* = 0.02, five studies, 372 women). There is no evidence for substantial statistical heterogeneity (chi^2^ = 7.31, *df* = 7 (*P* = 0.40); *I* ^2^ = 4 %) (Fig. 5). The number needed to treat for a benefit is 9 (95 % CI 5 to 33).

##### Secondary outcomes

3. Pregnancy
  There is no evidence for an effect in favor of the use of polyethylene oxide–sodium carboxymethylcellulose gel following operative hysteroscopy for suspected retained products of conception for the outcome of clinical pregnancy (RR 3.00, 95 % CI 0.35 to 25.68, *P* = 0.32, one study, 30 women) (Fig. 6).

**Fig. 6:**
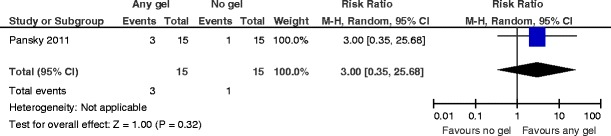
Any anti-adhesion gel versus no gel, outcome 3: pregnancy
4. Miscarriage
  There were no data for this secondary outcome.
5. Other secondary outcomes
  - Mean adhesion score at 3 months in women with fibroids, polyps, or uterine septa
    The use of auto-cross-linked hyaluronic acid gel in women undergoing operative hysteroscopy for myomas, endometrial polyps, or uterine septa is associated with a lower mean adhesion score at second-look hysteroscopy at 3 months (MD −1.44, 95 % CI −1.83 to −1.05, *P* < 0.00001, one study, 24 women). There is no evidence for substantial subgroup differences (chi^2^ = 0.24, *df* = 2 (*P* = 0.88), *I* ^2^ = 0 %) (Fig. 7).
  - Mean adhesion score at 3 months in women with intrauterine adhesions
    There are statistically significant differences in the lower mean adhesion scores at second-look hysteroscopy at 3 months in women undergoing operative hysteroscopy for intrauterine adhesions after the use of auto-cross-linked hyaluronic acid gel compared to operative hysteroscopy only (MD −3.30, 95 % CI −3.43 to −3.17, *P* < 0.00001, one study, 19 women) (Fig. 8).
  - Severity of adhesions at second-look hysteroscopy when using any gel
    At second-look hysteroscopy, there are more mild adhesions when using any gel following operative hysteroscopy (RR 2.81, 95 % CI 1.13 to 7.01, *P* = 0.03, four studies, 79 women). There is evidence for moderate statistical heterogeneity (chi^2^ = 12.30, *df* = 3 (*P* = 0.006); *I* ^2^ = 76 %) (Fig. 9). The number needed to treat to benefit is 2 (95 % CI 1 to 4).
    There is an effect favoring the use of any gel following operative hysteroscopy for the outcome of AFS 1988 stage II (moderate) adhesions at second-look hysteroscopy (RR 0.26, 0.09 to 0.80, *P* = 0.02, three studies, 58 women). There is no evidence for substantial statistical heterogeneity (chi^2^ = 1.43, *df* = 2 (*P* = 0.49); *I* ^2^ = 0 %) (Fig. 10). The number needed to treat to benefit is 2 (95 % CI 1 to 2).
    There is no evidence for a beneficial effect in favor of any gel versus no gel following operative hysteroscopy for the outcome of AFS 1988 stage III (severe) adhesions at second-look hysteroscopy (RR 0.46, 95 % CI 0.03 to 7.21, *P* = 0.58, three studies, 58 women) (Fig. 11). For the composite outcome “moderate or severe adhesions,” there are statistically significant differences favoring the use of any gel following operative hysteroscopy (RR 0.25, 95 % CI 0.10 to 0.67, *P* = 0.006, four studies, 79 women). There is no evidence for statistical heterogeneity (chi^2^ = 1.02, *df* = 3 (*P* = 0.80); *I* ^2^ = 0 %) (Fig. 12). The number needed to treat to benefit is 2 (95 % CI 1 to 4).

**Fig. 7:**
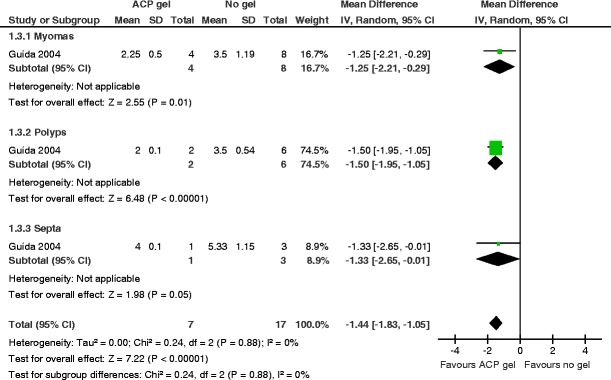
Auto-cross linked hyaluronic acid gel versus no gel, outcome 5.1: mean adhesion score AFS 1988 at 3 months in women with myomas, polyps, or uterine septa

**Fig. 8:**
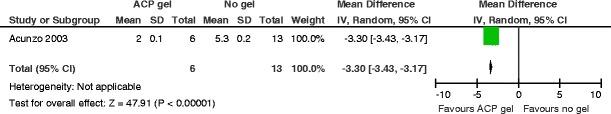
Auto-cross linked hyaluronic acid gel versus no gel, outcome 5.2: mean adhesion score AFS 1988 at 3 months in women with intrauterine adhesions

**Fig. 9:**
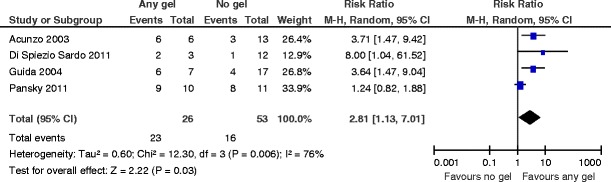
Any anti-adhesion gel versus no gel, outcome 5.3: AFS 1988 stage I (mild) adhesions at second-look hysteroscopy

**Fig. 10:**
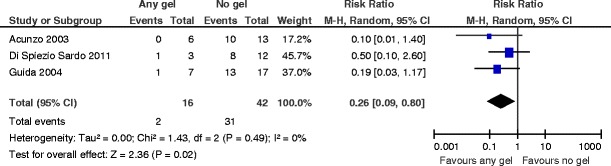
Any anti-adhesion gel versus no gel, outcome 5.3: AFS 1988 stage II (moderate) adhesions at second-look hysteroscopy

**Fig. 11:**
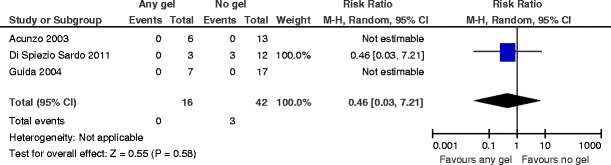
Any anti-adhesion gel versus no gel, outcome 5.3: AFS 1988 stage III (severe) adhesions at second-look hysteroscopy

**Fig. 12:**
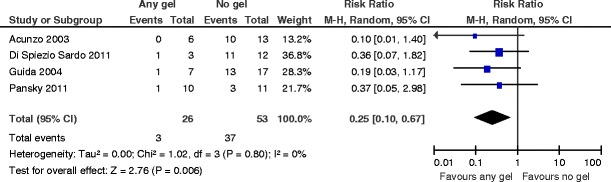
Any anti-adhesion gel versus no gel, outcome 5.3: AFS 1988 stage II (moderate) or stage III (severe) adhesions at second-look hysteroscopy

##### Subgroup analyses

Since no data were available for the outcome live birth, no subgroup analyses across studies reporting live birth and pregnancy rates or only one of these two key outcomes were done to assess any overestimation of treatment effect. There were enough data available to conduct a subgroup analysis according to the type of pathology treated by operative hysteroscopy. The use of any gel following operative hysteroscopy for fibroids (RR 0.44, 95 % CI 0.17 to 1.14, *P* = 0.09, two studies, 80 women), endometrial polyps (RR 0.32, 95 % CI 0.07 to 1.49, *P* = 0.15, two studies, 109 women), uterine septa (RR 0.26, 95 % CI 0.05 to 1.35, *P* = 0.11, two studies, 29 women), intrauterine adhesions (RR 0.44, 95 % CI 0.18 to 1.05, *P* = 0.06, one study, 84 women), retained products of conception (RR 0.91, 95 % CI 0.57 to 1.45, *P* = 0.69, one study, 30 women), or “not specified” (RR 0.87, 95 % CI 0.33 to 2.29, *P* = 0.78, one study, 40 women) consistently tends to decrease the incidence of de novo adhesions at second-look hysteroscopy; the differences were nevertheless not statistically significant given the limited numbers of women included and the limited numbers of events (Fig. 5). This is a common problem for subgroup analyses, and we should therefore be cautious about the interpretation of the data of this sensible predefined subgroup analysis. There is no evidence for substantial subgroup differences (chi^2^ = 5.69, *df* = 5 (*P* = 0.34), *I* ^2^ = 12.2 %).

##### Sensitivity analyses

A sensitivity analysis was done to study the impact of the study quality on the direction and magnitude of the treatment effect. If we excluded the single study at high risk of bias [37] in a sensitivity analysis, the use of any gel following operative hysteroscopy was still beneficial for decreasing the incidence of de novo adhesions, but the treatment effect was larger (RR 0.56, 95 % CI 0.35 to 0.90, *P* = 0.02, four studies, 332 women); there is no evidence for substantial statistical heterogeneity (chi^2^ = 7.39, *df* = 6 (*P* = 0.29); *I* ^2^ = 19 %) nor for substantial subgroup differences in the subgroup analysis according to the type of pathology treated (chi^2^ = 5.36, *df* = 4 (*P* = 0.25), *I* ^2^ = 25.4 %).

A sensitivity analysis to study the influence of the analysis model for data synthesis (fixed effect rather than random-effects model) did not influence the treatment effect (RR 0.55, 95 % CI 0.38 to 0.79, *P* = 0.001, five studies, 372 women). The choice of the summary effect measure (RR rather than OR) did not influence the treatment effect (OR 0.40, 95 % CI 0.23 to 0.70, *P* = 0.001, five studies, 372 women).

## Discussion

### Summary of main results

This systematic review aimed to appraise critically whether the use of anti-adhesion barrier gels following operative hysteroscopy for suspected or unsuspected intrauterine pathology in women of reproductive age suffering from infertility made a difference to the main outcomes of live birth, incidence of de novo adhesion formation, pregnancy, miscarriage, mean adhesions scores, or severity of adhesions at second-look hysteroscopy. We searched for RCTs on anti- adhesion barrier gels versus other barrier gels, placebo, or no anti-adhesion barrier gels following operative hysteroscopy.

We critically appraised five studies comparing the use of any anti-adhesion gel versus no gel in women of reproductive age treated by operative hysteroscopy for fibroids, polyps, septa, adhesions, or retained products of conception [36–40]. We judged a statistical pooling of the results of these five studies to be sensible given that substantial clinical diversity and statistical heterogeneity could confidently be ruled out.

According to our meta-analysis, there is evidence for an effect in favor of using anti-adhesive gel following operative hysteroscopy for decreasing the incidence of de novo adhesions at second-look hysteroscopy. By doing a predefined subgroup analysis, the consistency of this beneficial effect for this outcome could be demonstrated across different subgroups according to the type of pathology treated. The beneficial treatment effect for decreasing de novo adhesions at second-look hysteroscopy is robust as demonstrated by multiple sensitivity analyses evaluating the influence of study quality, choice of the analysis model for data synthesis, and the choice of the summary effect measure.

The use of auto-cross-linked hyaluronic acid gel in women undergoing operative hysteroscopy for fibroids, endometrial polyps, uterine septa, or intrauterine adhesions is associated with a lower mean adhesion score at second-look hysteroscopy at 3 months. When de novo adhesion formation is observed at second-look hysteroscopy, there are more mild adhesions and less moderate or severe adhesions by using any anti-adhesion gel after operative hysteroscopy.

There is no evidence for a treatment effect favoring the use of polyethylene oxide–sodium carboxymethylcellulose gel versus no gel in women treated by operative hysteroscopy for suspected retained products of conception for the outcome of pregnancy [40]. Although there was a beneficial trend when using the anti-adhesion gel, the differences between both comparison groups were not statistically significant. This may be a type II error: To detect a difference between both comparison groups of 13 % in the clinical pregnancy rate with a statistical power of 80 % at a confidence level of 95 % (*α* = 0.05 and *β* = 0.20), a sample size of 145 women would be needed instead of the much smaller number of 30 participants in this single center study. We refer to Table 1 for a summary of findings for the key outcomes clinical pregnancy and incidence of de novo adhesions at second-look hysteroscopy (Table 1).

**Table 1:** Summary of findings

Any anti-adhesive gel compared with no gel following operative hysteroscopy						
Patient or population: women of reproductive age treated by operative hysteroscopy for myomas, polyps, septa, adhesions, or retained products of conception						
Settings: hysteroscopy unit of a tertiary referral center						
Intervention: application of auto-cross linked hyaluronic acid or polyethylene oxide–sodium carboxymethylcellulose gel						
Comparison: no application of gel						
Outcomes	Illustrative comparative risks^a^ (95 % CI)		Relative effect (95 % CI)	No. of participants (studies)	Quality of the evidence (GRADE)	Comments: absolute effect
	Assumed risk control	Corresponding risk intervention				
Clinical pregnancy (time period not known)	Average risk population		RR 3.00 (0.35 to 25.68)	30 (1 study)	⊕⊝⊝⊝ (very low)	133 more per 1,000 (from 43 fewer to 1,645 more)
	67 per 1,000	201 per 1,000 (23 to 1,000)				
De novo adhesions at second look hysteroscopy (1 to 3 months)	Average risk population		RR 0.65 (0.45 to 0.93)	372 (5 studies)	⊕⊝⊝⊝ (very low)	102 fewer per 1,000 (from 20 fewer to 161 fewer)
	292 per 1,000	190 per 1,000 (131 to 272)				

The corresponding risk (and its 95 % confidence interval) is based on the assumed risk in the comparison group and the relative effect of the intervention (and its 95 % CI). GRADE Working Group grades of evidence: high quality—further research is very unlikely to change our confidence in the estimate of effect, moderate quality—further research is likely to have an important impact on our confidence in the estimate of effect and may change the estimate, low quality—further research is very likely to have an important impact on our confidence in the estimate of effect and is likely to change the estimate, and very low quality—we are very uncertain about the estimate

*CI* confidence interval, *RR* risk ratio

^a^The basis for the assumed risk is the pooled risk of the control groups of the five included studies [36–40]

### Overall completeness and applicability of evidence

The evidence of the effectiveness of using any anti-adhesion gel versus no gel in women of reproductive age treated by operative hysteroscopy for fibroids, polyps, septa, or intrauterine adhesions is limited: No data on live birth, pregnancy, or miscarriage rates were retrieved. In women of reproductive age treated by operative hysteroscopy for retained products of conception, the use of polyethylene oxide–sodium carboxymethylcellulose gel tends to increase the clinical pregnancy rate; the differences between both comparison groups were not statistically significant due to the small statistical power of the trial. Moreover, the proportion of the women suffering from infertility in both comparison groups was not reported, and the trial was at high risk of bias.

There are at the present two ongoing trials on the use of anti-adhesion barrier gels after operative hysteroscopy. The first is a parallel group randomized study on the effectiveness of applying Oxiplex/AP Gel (Intercoat) for preventing intrauterine adhesions in women aged 18 to 50 years following hysteroscopic surgery. This study is at the present not yet recruiting [41]. The second trial will address the effectiveness of hyaluronic acid gel in women older than 18 years following hysteroscopic surgery; this trial will not answer the research question in the present review since the primary and only outcome measured is the patient satisfaction rate 2 months after the gel application [42].

The applicability of the evidence retrieved is questionable; most trials were conducted in a target population including—but not limited to—women suffering from infertility: Two trials [36, 38] included variable proportions of women suffering from infertility, miscarriage, or risk of preterm delivery; for two studies [37, 39], it is unclear whether and how many participants suffered from infertility while the fifth study [40] included a source population of women with proven fertility trying to conceive after miscarriage. It is unlikely that the mechanisms whereby any of the studied interventions might decrease de novo adhesion formation might differ in infertile versus fertile target populations; nevertheless, we judge the overall applicability of the retrieved best available evidence in a more general source population to a target population of women suffering from infertility to be limited at the best.

### Quality of the evidence

We graded the evidence for the randomized comparison between any anti-adhesion barrier versus no gel following operative hysteroscopy for the outcome of pregnancy as very low. For this outcome, only one small study [40] was retrieved with few events. There are some methodological limitations: It is unclear whether and how allocation concealment and blinding of the outcome assessment were done in this study. Although lack of blinding of outcome assessors may be less relevant for an unequivocal outcome such as pregnancy, there might be some potential for risk of bias especially since the length of the follow-up period was not adequately described. The women included in this study were treated by operative hysteroscopy for retained products of conception following miscarriage; the proportion and the characteristics of individual women suffering from infertility were not described. The confidence intervals for the point effect estimate were moreover very wide. Formal study of reporting bias was not possible since only one study was retrieved for this outcome; this implies that reporting bias cannot be confidently ruled out.

For the outcome of incidence of de novo adhesions at second-look hysteroscopy, we graded the evidence as very low. We retrieved five studies in 372 women [36–40]. It was unclear whether and how allocation concealment and blinding of the outcome assessment were done in all studies. Lack of blinding of the outcome assessors is very relevant for this outcome since the interpretation of the presence and the hysteroscopic appearance of intrauterine adhesions is to some degree subjective. One study had serious methodological limitations due to high risk of attrition bias [37]. Less than 50 % of the participants of two of the five included studies were infertile women [36, 38] whereas it is unclear whether and how many women from the other three studies [37, 39, 40] suffered from infertility; this questions the applicability of the retrieved evidence in a more general source population including but not limited to women suffering from infertility to a target population of infertile women only. Although we could not formally investigate reporting bias given the small number of included studies, only studies demonstrating a beneficial effect were retrieved. Therefore, we judged that there might be some potential for reporting bias.

### Potential biases in the review process

Our group published a Cochrane review on the effectiveness of hysteroscopy in the treatment of female infertility associated with suspected major uterine cavity abnormalities [43]. Given our prior knowledge of potentially eligible studies, there might have been some potential for detection bias. We therefore aimed to conduct a comprehensive search strategy for the new clinical research question of the present systematic review; this has resulted in finding more studies than would have been detected using the previously developed search strategy.

### Agreements and disagreements with other studies or reviews

Two reviews support the use of anti-adhesive gel for reducing de novo adhesion formation following operative hysteroscopy. The first review [1] is a narrative review reporting the results and conclusions of one randomized trial included in the present systematic review [36]. The second review [44] is a systematic review and meta-analysis studying the effectiveness of auto-cross-linked hyaluronan gel for adhesion prevention in laparoscopic and hysteroscopic surgery. The data of three RCTs included in the present systematic review [36, 37, 39] were pooled: The proportion of women with adhesions at second look was significantly lower in women who received auto-cross linked hyaluronan gel than in the control group of women undergoing operative hysteroscopy without ACP gel (RR 0.50, 95 % CI 0.31 to 0.85, *P* = 0.009, three studies, 256 women). The authors used an older methodological tool (the Jadad scale) for assessing the validity of the included trials. The “scale” methodology is at the present no longer supported by the Cochrane Collaboration which recommends using a more formal assessment by means of the risk of bias tool. This different methodology explains the discrepancy between the statement of Mais et al. [44] that all the included trials in their systematic review were judged to be of a high quality which contrasts with our judgment of “very low quality evidence” for the outcomes of pregnancy and incidence of de novo adhesions.

One French small comparative study (*n* = 54 women with uterine pathology) studied the efficacy of auto-cross-linked hyaluronic acid gel in the prevention of adhesions following operative hysteroscopy [24]. Immediately after hysteroscopic surgery, the target population was divided by a non-random process into two groups: In group A, 30 women were treated by the intrauterine application of hyaluronic acid gel whereas the women in group B received no additional treatment (24 women). The key outcomes were the rate of adhesion formation, the mean adhesion score, and the adhesion severity according to the AFS classification, measured by second-look hysteroscopy 2 months after surgery. There are no statistically significant differences for the rate of intrauterine adhesion formation between the two groups (33.3 % for groups A and B) nor for the median adhesion scores (1.30 ± 2.35 versus 1.42 ± 2.47, *P* > 0.05) nor for the severity of the adhesions (70 % stage I adhesions, 20 % stage II adhesions, and 10 % stage III adhesions compared to 62.5 % stage I, 25 % stage II, and 12.5 % stage III in groups A and B, respectively, *P* > 0.05). The authors conclude that the use of auto-cross-linked hyaluronic acid gel does not reduce the incidence and the severity of intrauterine adhesions after hysteroscopic surgery. According to a more recent review of the literature [45]—with the first author of the French comparative study [24] as co-author—the majority of the limited published studies until 2008 only evaluated the anatomic efficiency of anti-adhesion agents after hysteroscopic surgery. The authors conclude that the available data for the key reproductive outcomes are not sufficiently convincing to promote the widespread clinical use of anti-adhesive barrier agents as an effective treatment strategy for infertile women treated by operative hysteroscopy, hence their conclusion that additional randomized controlled trials are needed.

## Authors’ conclusions

### Implications for practice

Gynecologists should counsel their patients that intrauterine adhesion formation is the major long-term complication of operative hysteroscopy in women of reproductive age. They might consider using any barrier gel following operative hysteroscopy for suspected uterine cavity abnormalities in infertile women: Its use may decrease de novo adhesion formation (very low quality evidence). If de novo adhesion formation occurs, there are less moderate or severe adhesions and more mild adhesions by using any anti-adhesion gel; the mean adhesion scores at second-look hysteroscopy are lower after using ACP gel. Infertile women nevertheless should be counseled that there is no evidence for higher live birth or pregnancy rates by using any barrier gel following operative hysteroscopy (very low-quality evidence). There are no data at the present of the effects on the miscarriage rates.

### Implications for research

The very low-quality evidence retrieved from the limited number of randomized studies in a general source population including, but not restricted to, infertile women is at the present not sufficient to draw robust conclusions in favor of any barrier as an adjunctive therapy following operative hysteroscopy for the key reproductive outcomes; more well-designed pragmatic RCTs are needed to assess whether the use of any anti-adhesion gel affects the live birth, the pregnancy, and miscarriage rates in a target population of infertile women. There are no data on a dose–response relationship between the size, the number, or the severity of the treated pathology and the corresponding magnitude of the increase in effectiveness or decrease in the adverse outcomes that were defined in the present systematic review.
